# The Antioxidant N-Acetylcysteine Prevents HIF-1 Stabilization under Hypoxia *In Vitro* but Does Not Affect Tumorigenesis in Multiple Breast Cancer Models *In Vivo*

**DOI:** 10.1371/journal.pone.0066388

**Published:** 2013-06-20

**Authors:** Jaclyn Sceneay, Mira C. P. Liu, Anna Chen, Christina S. F. Wong, David D. L. Bowtell, Andreas Möller

**Affiliations:** 1 Cancer Genomics and Genetics Laboratory, Peter MacCallum Cancer Centre, East Melbourne, Victoria, Australia; 2 Department of Pathology, The University of Melbourne, Parkville, Victoria, Australia; 3 Tumour Microenvironment Laboratory, Queensland Institute of Medical Research, Herston, Queensland, Australia; 4 Sir Peter MacCallum Department of Oncology, The University of Melbourne, Parkville, Victoria, Australia; University of Melbourne, Australia

## Abstract

Intratumoral hypoxia is a poor prognostic factor associated with reduced disease-free survival in many cancer types, including breast cancer. Hypoxia encourages tumor cell proliferation, stimulates angiogenesis and lymphangiogenesis, and promotes epithelial-mesenchymal transition and metastasis. Tumor cells respond to a hypoxic state by stabilizing the Hif-1α subunit of the Hypoxia-Inducible Factor (HIF) transcription factor to promote expression of various tumor- and metastasis-promoting hypoxic response genes. The antioxidant N-acetylcysteine (NAC) was recently shown to prevent Hif-1α stabilization under hypoxia, and has been identified as a potential alternative method to target the hypoxic response in tumors. We utilized three orthotopic syngeneic murine models of breast cancer, the PyMT, EO771 and 4T1.2 models, to investigate the ability of NAC to modulate the hypoxic response *in vitro* and *in vivo*. While NAC prevented Hif-1α stabilization under hypoxia *in vitro* and increased levels of glutathione in the blood of mice *in vivo*, this did not translate to a difference in tumor growth or the hypoxic state of the tumor compared to untreated control mice. In addition, NAC treatment actually increased metastatic burden in an experimental metastasis model. This work raises questions regarding the validity of NAC as an anti-tumorigenic agent in breast cancer, and highlights the need to further investigate its properties *in vivo* in different cancer models.

## Introduction

Hypoxic conditions in tumors correlate with a poor prognosis, reduced disease-free survival and overall survival in cancer patients [1], [2]. Hypoxia refers to conditions in solid tumors where the oxygen pressure is less than 5–10 mm Hg, which initially occurs when the vasculature of the local environment is unable to meet the needs of rapidly proliferating tumor cells, and persists due to aberrant tumor vasculature [2]–[5]. Neo-angiogenesis, as well as enhanced proliferation, anabolic metabolism, lymphangiogenesis, epithelial-mesenchymal transition (EMT) and suppression of immune surveillance are consequences of tumor hypoxia [6]. Hypoxic tumors are also more resistant to chemotherapy and radiotherapy [7], [8].

The cellular response to hypoxia is largely mediated by formation of the hypoxia-inducible factor (HIF) transcription factor, a basic helix-loop-helix heterodimeric protein consisting of the oxygen-dependent α and constitutively expressed β subunits [9]–[14]. Binding of the HIF transcription factor to hypoxic-response elements (HREs) promotes the expression of genes involved in glycolysis, angiogenesis, cell survival and proliferation, invasion and metastasis [2], [15]–[17]. Hif-1α and Hif-2α are the major α-subunit isoforms, with Hif-1α being the master regulator of the transcriptional cellular response to hypoxia [2]. Increased Hif-1α protein levels in primary tumor biopsies correlates with poor outcome in breast, ovarian, pancreatic, bladder, colorectal and lung cancer among others (reviewed in [18]). Under normoxic conditions, Hif-1α is hydroxylated at conserved proline residues, Pro402 and Pro564, by oxygen ‘sensing’ prolyl hydroxylases (PHDs), creating a recognition signal for the von Hippel-Lindau (pVHL) ubiquitin ligase complex. Subsequent polyubiquitination by pVHL marks Hif-1α for degradation by the 26S proteasome [19]–[25].

Numerous drugs have been used to target different aspects of the hypoxic signaling pathway to inhibit tumor growth, angiogenesis and metastasis in mouse models. These drugs include anthracyclines such as doxorubicin, as well as inhibitors of histone-deacteylases (HDACs), heat shock protein (HSP)-90, mTOR and EGFR [26]–[31]. Some drugs have progressed successfully to the clinic such as topotecan, a topoisomerase I inhibitor [32], suggesting that targeting hypoxic signaling is a promising approach for cancer therapy.

Recently, the antioxidant N-acetylcysteine (NAC) was identified as a novel inhibitor of the hypoxic response pathway [33]. NAC has been approved by the FDA in the treatment of chronic obstructive lung disease (COPD) and as an antidote to acetaminophen (paracetamol) overdose [34], [35]. It is a promiscuous antioxidant with many different clinical applications, ranging from the treatment of contrast-induced nephropathy to influenza and idiopathic pulmonary fibrosis [35]. NAC is a precursor of glutathione (GSH), the body’s major antioxidant, and supplementation with NAC increases GSH levels to support the antioxidant and nitric oxide systems during infection, inflammation, stress and exposure to toxins [35]–[37]. NAC has also been trialled as an anti-tumorigenic agent due to its ability to remove reactive oxygen species (ROS), which promote tumorigenesis by inducing DNA damage and genomic instability [37], [38]. Anti-tumorigenic effects of NAC as a single agent have been demonstrated in B16-BL6 melanoma [39], [40], Kaposi’s sarcoma [37] and MDA-MB-435 xenograft breast cancer models [36]. NAC has also been shown to act synergistically with doxorubicin in melanoma to reduce tumor growth and metastasis [39], [40]. More recently, the anti-cancer effects of NAC in P493 human B cell xenografts in SCID mice and in a Myc-dependent transgenic model of hepatocellular carcinoma were shown to be highly dependent on HIF-1 [33].

Cells undergoing hypoxic stress produce increased amounts of ROS [41], which enhance tumorigenesis by contributing to Hif-1α stabilization. ROS are capable of inactivating PHDs, in particular PHD2, through oxidation of the ferrous ion essential for the catalytic hydroxylation of prolines, rendering them unable to hydroxylate Hif-1α under hypoxia [33], [42]. Reducing ROS through the administration of NAC was shown to recover PHD2 activity and prevent Hif-1α stabilization under hypoxia [33]. Several studies have also indirectly linked the anti-tumorigenic activity of NAC to its modulation of the hypoxic response. In both Kaposi’s sarcoma and xenograft breast cancer models, NAC treatment decreased tumorigenesis by inhibiting the angiogenic response in tumor cells [36], [37], while in a B16-BL6 melanoma model, NAC selectively inhibited type-IV collagenases including MMP2 and MMP9 [39], [43], key HIF-1 target genes involved in invasion and metastasis [1]. In addition, NAC has been demonstrated to reduce metastasis by preventing hypoxia-induced EMT of tumor cells in pancreatic cancer [44].

In this study, we investigated the ability of NAC to alter tumor growth and metastasis in a HIF-dependent manner in multiple orthotopic, syngeneic murine models of breast cancer. Using the PyMT, EO771 and 4T1.2 breast cancer models, each with different metastatic capacities, we showed that while NAC prevents Hif-1α stabilization *in vitro*, it does not reduce primary tumor growth and that it, in fact, increases the number of metastatic foci in an experimental metastasis model, raising the question of whether NAC is truly beneficial in the treatment of breast cancer.

## Materials and Methods

### Ethics Statement

All animal procedures were conducted in accordance with Australian National Health and Medical Research regulations on the use and care of experimental animals, and approved by the Peter MacCallum Cancer Centre Animal Experimentation Ethics Committee (E452).

### Cell Lines

The C57Bl/6 PyMT mammary tumor cell line was derived from a primary tumor in a female C57Bl/6 MMTV-PyMT mouse and the establishment and maintenance conditions have been previously described [45], [46]. The generation of the C57Bl/6 EO771 [47] and BALB/c 4T1.2 [48] mammary carcinoma cell lines have been detailed and the cell lines were maintained as previously described [46], [49], [50].

### *In vitro* Hif-1α Staining

For cellular Hif-1α staining, PyMT and EO771 tumor cells were plated on glass slides and stained with anti-Hif-1α antibody (Novus Biologicals), co-stained with diaminobenzidine-stained (DAB) and images taken on a BX-51 microscope (Olympus).

### Western Blot

PyMT and EO771 cell lysates were separated on a 10% acrylamide gel and protein abundance analyzed by Western blotting as previously described [49]. EO771 tumors were weighed and equal amounts homogenized in protein lysis buffer (2% SDS, 50 mM Tris, pH 6.85) to generate tumor protein lysates as described previously [45]. Membranes were probed for Hif-1α (Novus Biologicals), Hif-2α (Novus Biologicals), E-Cadherin (BD Pharmingen), β-actin (Sigma) and α-tubulin (Sigma) and detected using the enhanced chemiluminescence system (GE Healthcare).

### ELISA

VEGF-A concentration was measured in the supernatant of PyMT and EO771 tumor cells exposed to normoxic (20% O_2_) or hypoxic (2% O_2_) conditions for 8 hours using the mouse VEGF Quantikine ELISA Kit (R&D Systems) according to the manufacturer’s instructions.

### Real Time PCR

mRNA from cells was isolated by QiaShredder (Qiagen) and RNeasy (Qiagen) kits according to the manufacturer’s instructions. Mouse primer sequences for VEGF are 5′-CCATGCAGATCATGCGGATCA-3′ and 3′-CCTTGGCTTGTCACATCTGCAA-5′; LOX 5′-TCTTCTGCTGCGTGACAACC-3′ and 3′-GAGAAACCAGCTTGGAACCAG-5′; SLUG 5′-TGGTCAAGAAAC ATTTCAACGCC-3′ and 3′- GGTGAGGATCTCTGGTTTTGGTA; SNAIL 5′- CACACGCTGCCTTGTGTCT-3′ and 3′- GGTCAGCAAAAGCACGGTT-5′; TWIST 5′- GGACAAGCTGAGCAAGATTCA-3′ and 3′- CGGAGAAGGC GTAGCTGAG-5′; β2M (housekeeping control) 5′-TTCACCCCCACTGAGACTG AT-3′ and 3′-GTCTTGGGCTCGGCCATA-5′ (Geneworks).

### Mice

C57Bl/6 mice were bred and maintained at the Peter MacCallum Cancer Centre (Melbourne, Australia). BALB/C mice were purchased from the Walter and Eliza Hall Institute (Melbourne, Australia). Female mice 8–14 weeks of age were used for all experiments and cohort sizes of five or more mice per experimental group used as described for each experiment.

### *In vivo* Primary Tumor Models

For primary tumor growth, mice were orthotopically injected with 2×10^5^ EO771 and 4T1.2 or 5×10^5^ PyMT mammary tumor cells resuspended in 20 µl PBS into the 4^th^ left mammary fat pad under anesthetic (5% ketamine; 1% xylazil (Ilium) solution). Tumor growth was monitored every third day using digital calipers and mice culled by anesthetic inhalation overdose (isoflurane; Aerrane) when the tumor volume reached a total of 525 mm^3^ (calculated as π × length × width^2^/6), defined as endstage for primary tumor growth experiments and survival analysis in accordance with animal ethics.

### *In vivo* Metastasis Models

For the PyMT and 4T1.2 primary-resection models, the primary tumor was surgically resected from the mammary fatpad at a tumor volume of 33.5 mm^3^ under anaesthetic (5% ketamine; 1% xylazil (Ilium) solution) and mice culled by anesthetic inhalation overdose (isoflurane; Aerrane) 10 weeks post-resection or at metastatic endstage (obvious panting, inactivity, ruffled fur, limping or mice have lost greater than 20% of their body weight compared to the start of the experiment). For experimental metastasis models, 2×10^5^ EO771 tumor cells in 100 µl PBS were intravenously injected via the tail vein and mice culled 2 weeks post-injection.

### N-acetylcysteine Treatment

*In vitro*, NAC was added at 10 mM, 25 mM or 40 mM to cells under normoxic (20% O_2_) or hypoxic conditions (2% O_2_) for time points indicated. *In vivo*, NAC was administered at 40 mM into the drinking water of the mice daily as previously described [33] for times indicated until endpoint.

### Immunofluorescence

Serial sections were obtained from optimal cutting temperature (OCT) (fresh frozen)-imbedded tumors. Sections were post-fixed with acetone and stained with antibodies for CD31 (BD Biosciences Pharmingen) and Hif-1α (Novus Biologicals) as described [45], [49]. Images were taken on a BX-51 microscope (Olympus), using SpotAdvanced software. Five random fields were imaged per section at 20x magnification, and five sections per tumor (total 25 fields per tumor) analyzed. Thresholds for positive CD31 or Hif-1α signals were determined by subtracting background signals obtained from isotype controls. Using the same threshold for each image, data was analyzed using Metamorph software in a semi-automated manner. For detection of hypoxic regions within primary tumors, mice with endstage tumors were injected with 60 mg/kg pimonidazole (PIM) via the tail vein and tumors dissected and frozen in OCT solution 3 hours later. PIM was detected in OCT-embedded tumors using Hypoxyprobe Green as described [51].

### Immunohistochemistry

Serial sections were obtained from 10% neutral-buffered formalin (NBF)-fixed, paraffin embedded (FFPE) tumors or lungs. To assess whole tumor morphologies or count lung metastatic foci, sections were stained with hemotoxylin and eosin (H&E). Tumor sections were scanned using Aperio Imaging Software, and lung metastatic foci counted under a dissecting microscope on whole-lung sections. Four sections per lung were counted to obtain an average number of metastatic foci. Proliferation in lung FFPE sections were assessed by incubation with a Ki67 antibody (Cell Marque) and visualization with DAB as detailed previously [45]. Sections were counterstained with hematoxylin. Ki67 staining was analyzed using the ImmunoRatio automated image analysis program for Ki67 immunostained tissue [52]. The program segments DAB and hematoxylin-stained nuclei regions from the image, calculates a labeling index of the DAB-stained nuclear area as a percentage of total nuclear area, and generates a pseudo-colored result image matching the area segmentation (examples in Figure S1B).

Apoptotic cells were detected using the Apoptag Peroxidase In Situ Apoptosis Detection Kit (Chemicon International) according to the manufacturer’s instructions, and visualized with DAB. Sections were then counterstained with hemotoxylin. Each metastatic tumor from a minimum of six mice per group was assessed independently (n = 1). Abundance of apoptotic cells (negative, one, two or three plus) was determined in a blinded fashion by CSFW and AM according to examples shown in Figure.

### Glutathione Measurements

Total glutathione (GSH) was measured in both blood and tumors of control and NAC-treated mice using the Bioxytech GSH/GSSG-412 kit (OxisResearch) according to the manufacturer’s instructions. For blood, 50 µl was obtained by eye bleeding mice at different time points after NAC treatment. Tumors were obtained at dissection and frozen at −80°C, weighed and homogenized in 5% metaphosphoric acid to obtain the concentration of GSH (nmol/mg).

### Statistical Analysis

Results are expressed as mean ± SEM and analyzed by unpaired Student’s two-tailed t-Tests. Kaplan-Meier Survival curves were analyzed using Log-rank (Mantel-Cox) Test. p values <0.05 were considered significant (***p<0.0001, **p<0.01 and *p<0.05).

## Results

### NAC Prevents Hif-1α Stabilization in vitro

**Figure 1:**
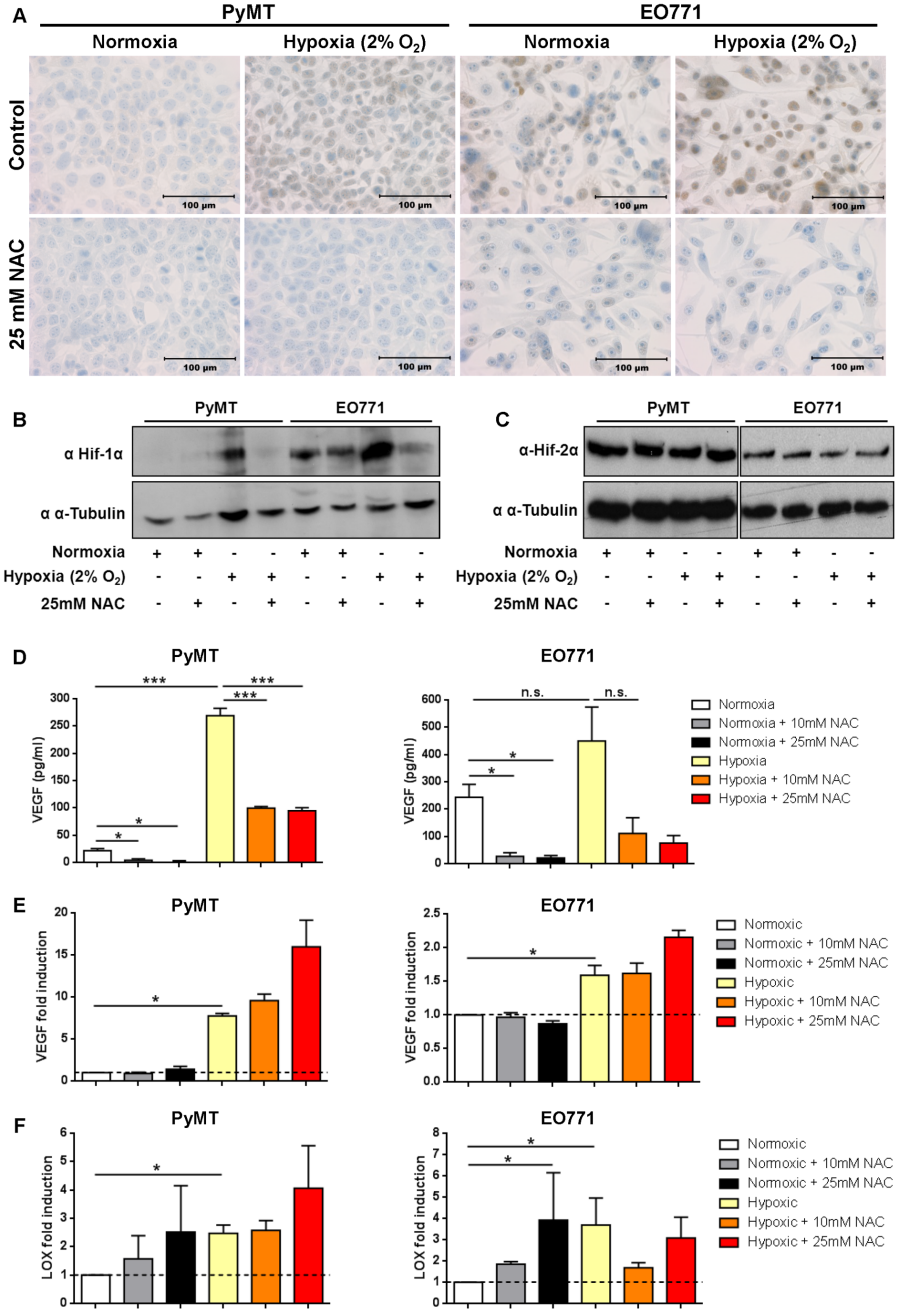
*In vitro* characterization of NAC treatment on the hypoxic response pathway. **A)** Representative images of PyMT and EO771 cells exposed to normoxic (20% O_2_) or hypoxic (2% O_2_) conditions for 2 hours and treated with 0 (control) or 25 mM NAC. Cells were fixed with 10% NBF and stained for Hif-1α and DAB (positive cells are stained brown). Scale bars represent 100 µm. **B-C)** Cell lysates from PyMT and EO771 cells exposed to normoxic and hypoxic conditions for 2 hours and treated with 0 or 25 mM NAC, and probed for Hif-1α **(B)**, Hif-2α **(C)** and α–tubulin (loading control) by Western blot. **D)** Secreted VEGF-A (pg/mL) was measured by ELISA in the supernatant of PyMT and EO771 cells exposed to normoxic or hypoxic conditions for 8 hours with 0, 10 or 25 mM NAC. p value for EO771 is 0.174, n = 4. **E–F)** mRNA was extracted from PyMT and EO771 cells exposed to normoxic or hypoxic conditions for 8 hours and treated with 0, 10 or 25 mM NAC. Expression of hypoxic response genes VEGF **(E)** and LOX **(F)** was assessed using qRT-PCR and fold induction normalized to normoxic sample (broken line; n = 3 in triplicate). Mean ± SEM; n.s. indicates not significant; *p<0.05; ***p<0.001; Student’s t-Test.

To examine the effect of NAC on the hypoxic response *in vitro*, PyMT and EO771 breast tumor cell lines were exposed to normoxic (20% O_2_) or hypoxic (2% O_2_) conditions. Hif-1α was stabilized in cells exposed to hypoxia compared to normoxia in both cell lines as detected by cellular staining (Figure 1A) and Western blotting (Figure 1B). Treatment with 25 mM NAC reduced Hif-1α stabilization under hypoxic conditions in both cell lines, and under normoxic conditions in EO771 cells, which show a higher baseline level of Hif-1α expression (Figure 1A-B). NAC treatment did not change the abundance of Hif-2α under hypoxia in either cell line (Figure 1C), indicating NAC only prevents stabilization of Hif-1α. VEGF, one of the main hypoxic response genes and a key promoter of angiogenesis, was also reduced in the supernatant of hypoxic EO771 and PyMT cells after NAC treatment as detected by ELISA (Figure 1D). Interestingly, although VEGF gene expression was increased under hypoxia in both PyMT and EO771 cells, treatment with 10 mM and 25 mM NAC actually increased VEGF gene expression under hypoxia (Figure 1E). LOX, a HIF-1 target gene that promotes invasion and metastasis, was also increased under hypoxia compared to normoxia, but remained unchanged or slightly increased after treatment with 10 mM and 25 mM NAC under hypoxia (Figure 1F). NAC treatment was therefore able to prevent the stabilization of Hif-1α and reduce VEGF secretion in response to hypoxia in both PyMT and EO771 breast tumor cells *in vitro*, but not alter the hypoxia-induced gene expression of VEGF and LOX.

### NAC does not Prevent Transcription of the EMT-target Gene TWIST under Hypoxia

**Figure 2:**
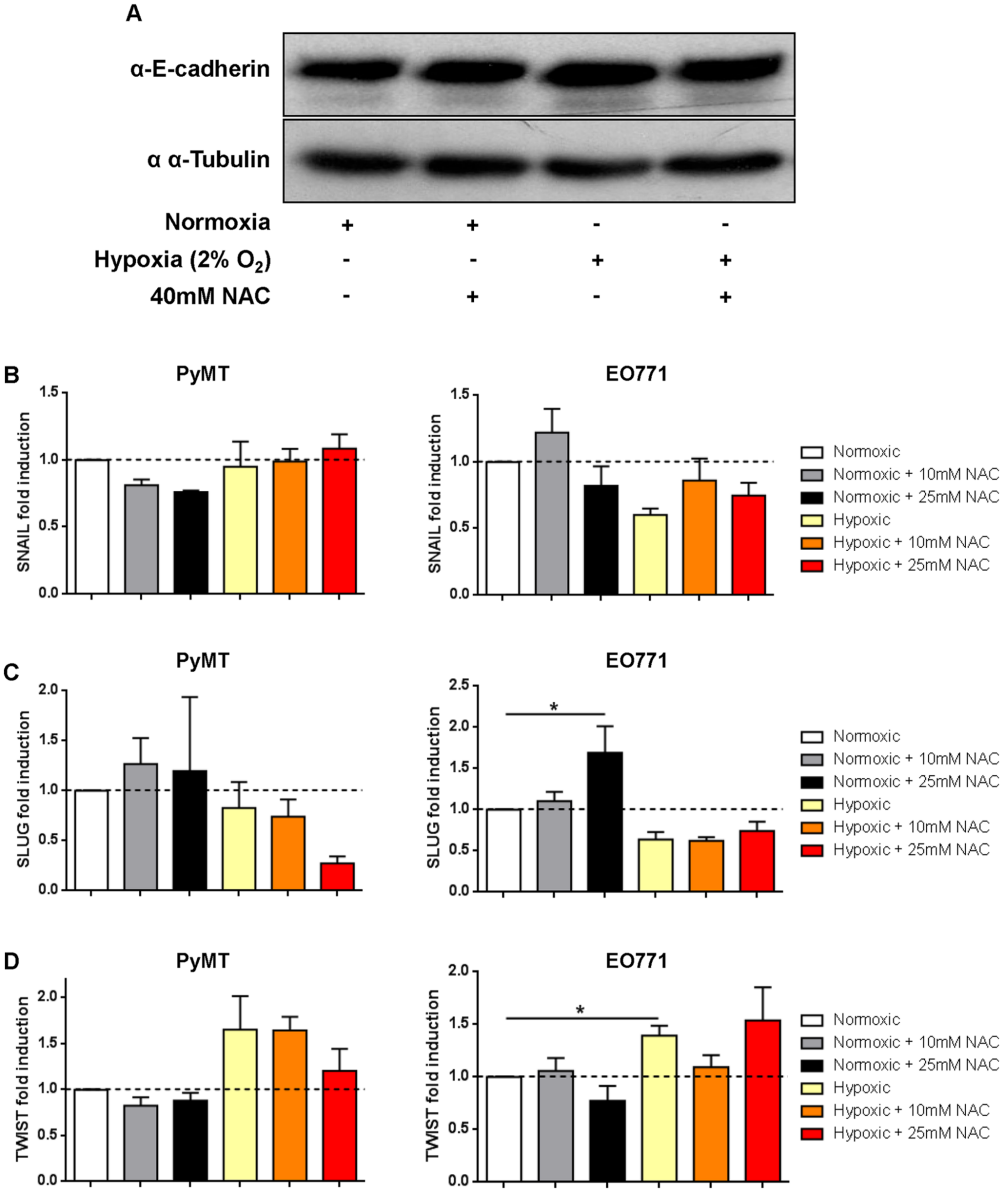
NAC treatment does not prevent EMT under hypoxia *in vitro*. **A)** Cell lysates from PyMT cells exposed to normoxic and hypoxic conditions for 2 hours and treated with 40 mM NAC and probed for E-cadherin and α–tubulin (loading control) by Western blot. **B–D)** mRNA was extracted from PyMT and EO771 cells exposed to normoxic or hypoxic conditions for 8 hours with 0, 10 or 25 mM NAC treatment. Expression of EMT target genes SNAIL **(B)**, SLUG **(C)** and TWIST **(D)** was quantified using qRT-PCR and fold induction normalized to normoxic sample (broken line; n = 3 in triplicate). Mean ± SEM; *p<0.05; Student’s t-Test.

The impact of NAC on hypoxia-induced EMT was assessed by E-cadherin protein abundance using Western blotting. No change in E-cadherin expression was observed between NAC-treated and control cells (Figure 2A). Of the major EMT transcription factors, SNAIL, SLUG and TWIST, only TWIST mRNA levels were increased under hypoxia in EO771 cells, but not PyMT cells (Figure 2B-D). Treatment with 10 mM and 25 mM NAC failed to reduce TWIST mRNA levels under hypoxia (Figure 2D). Therefore, while hypoxia induces minor changes in the EMT transcription factor TWIST, NAC treatment did not inhibit this response *in vitro*.

### NAC Treatment Increases GSH Levels in Circulation

**Figure 3:**
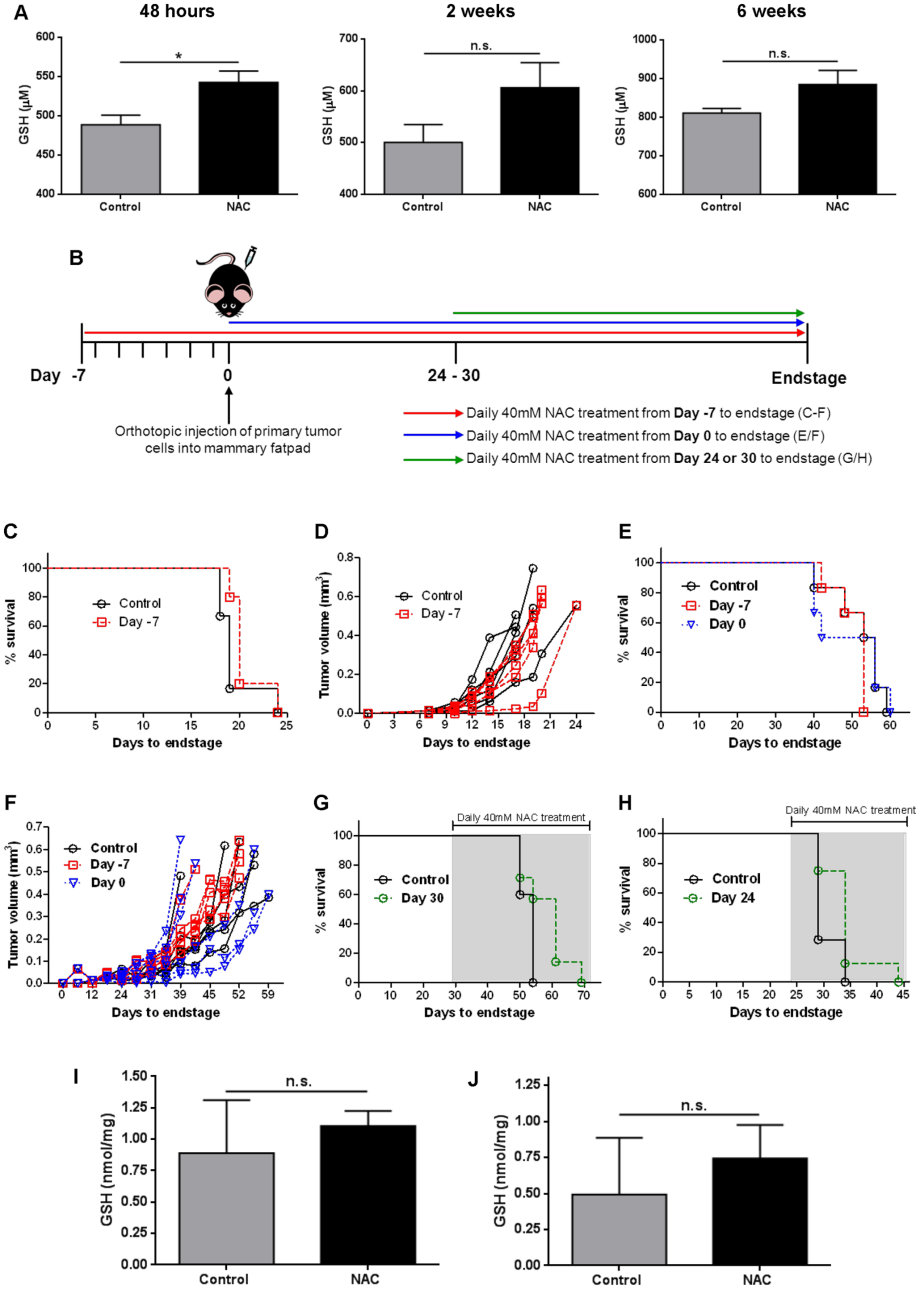
NAC increases GSH levels in blood but does not affect primary tumor growth *in vivo*. **A)** **** GSH (µM) was measured in 50 µl blood taken from control mice or mice treated with 40 mM NAC in the drinking water for 48 hours (n = 4 per group), 2 weeks (n = 4−5 per group) or 6 weeks (n = 6 per group). Mean ± SEM; *p<0.05; Student’s t-Test. **B)** Schematic of NAC treatment regimens for primary tumor growth. PyMT, EO771 or 4T1.2 breast tumor cells were orthotopically injected on day 0, and 40 mM NAC treatment administered daily from day -7 prior to tumor cell injection (red line), on day 0 (blue line) or at day 24–30 (green line) until endstage (tumor volume 525 mm^3^). Control mice were injected with tumor cells but did not receive NAC treatment. **C)** Kaplan-Meier survival curve for mice treated with 40 mM NAC from day -7 prior to EO771 tumor cell injection and control mice (black line) (control n = 6; Day -7 NAC n = 5; p = 0.18). **D)** Tumor growth curves for each individual mouse (shown as tumor volume in mm^3^) from animals in C. **E)** Kaplan-Meier survival curve for mice treated with 40 mM NAC from day -7 prior to PyMT tumor cell injection or day 0, and controls (n = 6 per group; p = 0.67). **F)** Tumor growth curves for each individual mouse (tumor volume in mm^3^) from E. **G)** Kaplan-Meier survival curve of mice treated with 40 mM NAC from day 30 after PyMT tumor cell injection and controls (control n = 7; Day 30 NAC n = 8; p = 0.10). **H)** Kaplan-Meier survival curve of mice treated with 40 mM NAC from day 24 after 4T1.2 tumor cell injection and controls (control n = 7, Day 24 NAC n = 8; p = 0.07). p values calculated using Log-rank (Mantel-Cox) Test. **I–J)** Concentration of GSH (nmol/mg) in EO771 tumors **(I)** from animals in C (control n = 3; NAC n = 5), and 4T1.2 tumors **(J)** from animals in H (n = 6 for all groups). n.s. indicates not significant.

Next, we sought to determine the impact of NAC *in vivo.* A previous report demonstrated that drinking water supplemented with 40 mM NAC was sufficient to impact tumor growth in a MYC-dependent B cell lymphoma model [33]. As NAC is a precursor of GSH, NAC supplementation should increase GSH levels. Mice that were supplemented with 40 mM NAC in their drinking water had significantly increased GSH levels in their blood after 48 hours of treatment (Figure 3A). While GSH levels in these mice remained elevated after 2 weeks and 6 weeks, these increases were not significant (p = 0.11 and p = 0.09 respectively).

### NAC Treatment does not Alter Primary Tumor Growth in vivo

To assess the effect of NAC treatment on primary mammary tumor growth, three orthotopic, syngeneic murine models (PyMT, EO771 and 4T1.2) were used. PyMT cells, derived from a spontaneous Polyoma-Middle T mammary gland tumor [45], are slower growing and have a low metastatic capacity compared to the highly aggressive EO771 [49] and 4T1.2 [50] cell lines. Three NAC treatment schedules were devised as depicted in Figure 3B. NAC (40 mM) was administered to the drinking water either 7 days prior to primary cell injection (day -7), at the time of injection (day 0) or once the primary tumor was established (tumor volume of 250 mm^3^). Pre-treatment with NAC from day -7 did not change survival (Figure 3C; endstage tumor volume of 525 mm^3^) or tumor growth rate (Figure 3D) compared to untreated control mice in the EO771 model (control n = 6; NAC n = 5). Similarly, in the PyMT model, pre-treatment with NAC from day -7 or day 0 did not change survival or tumor growth rates (Figure 3E–F; n = 6 for each group). Furthermore, NAC treatment did not change overall survival in mice with established PyMT tumors (Figure 3G; control n = 7; NAC n = 8) or 4T1.2 tumors (Figure 3H; control n = 7; NAC n = 8). Therefore, regardless of the timing of treatment, NAC provided no survival benefits and did not alter tumor growth rates.

### NAC Treatment does not Increase GSH Levels in Primary Breast Tumors

**Figure 4:**
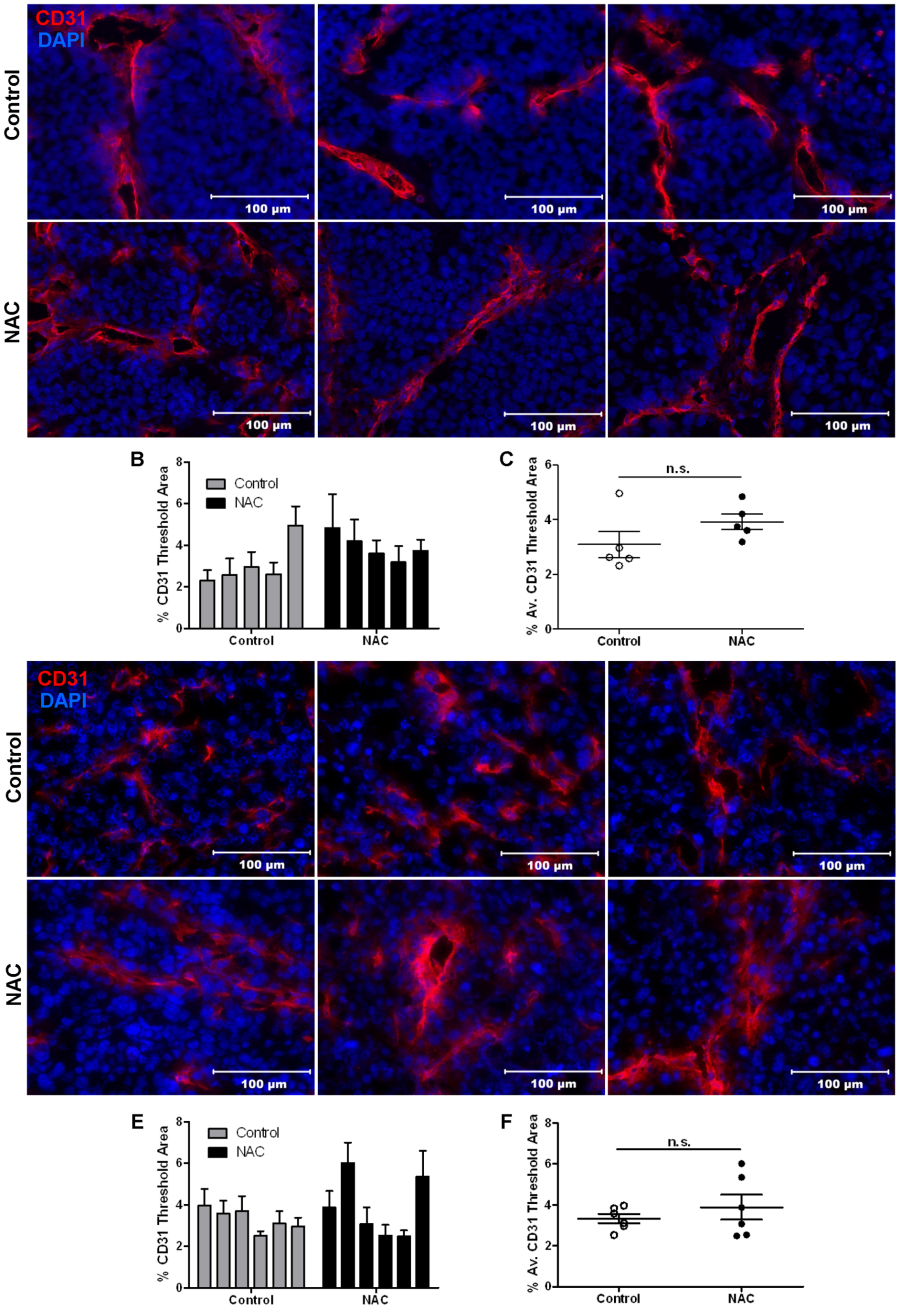
NAC treatment does not alter the angiogenic phenotype of primary tumors. Tumors from NAC-treated or control mice were stained with CD31 (red) and DAPI (blue). Five sections per tumor were stained and imaged to obtain an average percentage threshold area of CD31 staining determined from 5 random images per section. **A)** Representative images of PyMT tumors (from Figure 3G) with CD31 staining values calculated for individual tumors (**B)** and averaged **(C)** (n = 5 tumors for all groups). **D)** Representative images of EO771 tumors (from Figure 3C–D) with CD31 staining values calculated for individual tumors (**E)** and averaged **(F)** (n = 6 tumors for all groups). Scale bars represent 100 µm. Mean ± SEM; n.s. indicates not significant.

Next we investigated if the increase in GSH levels observed in the circulation of NAC-treated mice (Figure 3A) resulted in increased GSH levels in tumors. The total GSH levels was measured in EO771 tumors from mice pre-treated with NAC from day -7 (from Figure 3C), or 4T1.2 tumors from mice with established tumors at the time of NAC treatment (from Figure 3H), and normalized to the weight (mg) of the tumor. The resulting GSH concentration (nmol/mg) varied in control tumors, but overall there was no significant difference when compared to NAC tumors in both EO771 (Figure 3I) and 4T1.2 (Figure 3J) models (p = 0.55 and p = 0.21 respectively).

### NAC Treatment does not change the Angiogenic Phenotype of Breast Tumors

To investigate whether NAC treatment would alter neo-angiogenesis based on its effects on VEGF secretion (Figure 1D), tumors from NAC-treated and control mice were stained for the endothelial cell marker CD31. Quantification of blood vessels showed no difference in CD31-positive vessels in PyMT tumors from mice treated with NAC after establishment of the primary tumor compared to controls (Figure 4A–C; control n = 7; NAC n = 8; 5 sections stained and analyzed per tumor (n)). Furthermore, no difference in overall necrosis was observed in whole sections of PyMT tumors from mice treated with NAC after establishment of the primary tumor (Figure S1A). Pre-treatment with NAC from day -7 before EO771 tumor cell injection also had no effect on the CD31-positive vessel abundance (Figure 4D–F; n = 6 for each group; 5 sections stained and analyzed per tumor). Therefore, treatment with NAC did not alter vessel density or necrosis in primary breast tumors.

### NAC Treatment does not Change the Hypoxic Phenotype in Breast Tumors

**Figure 5:**
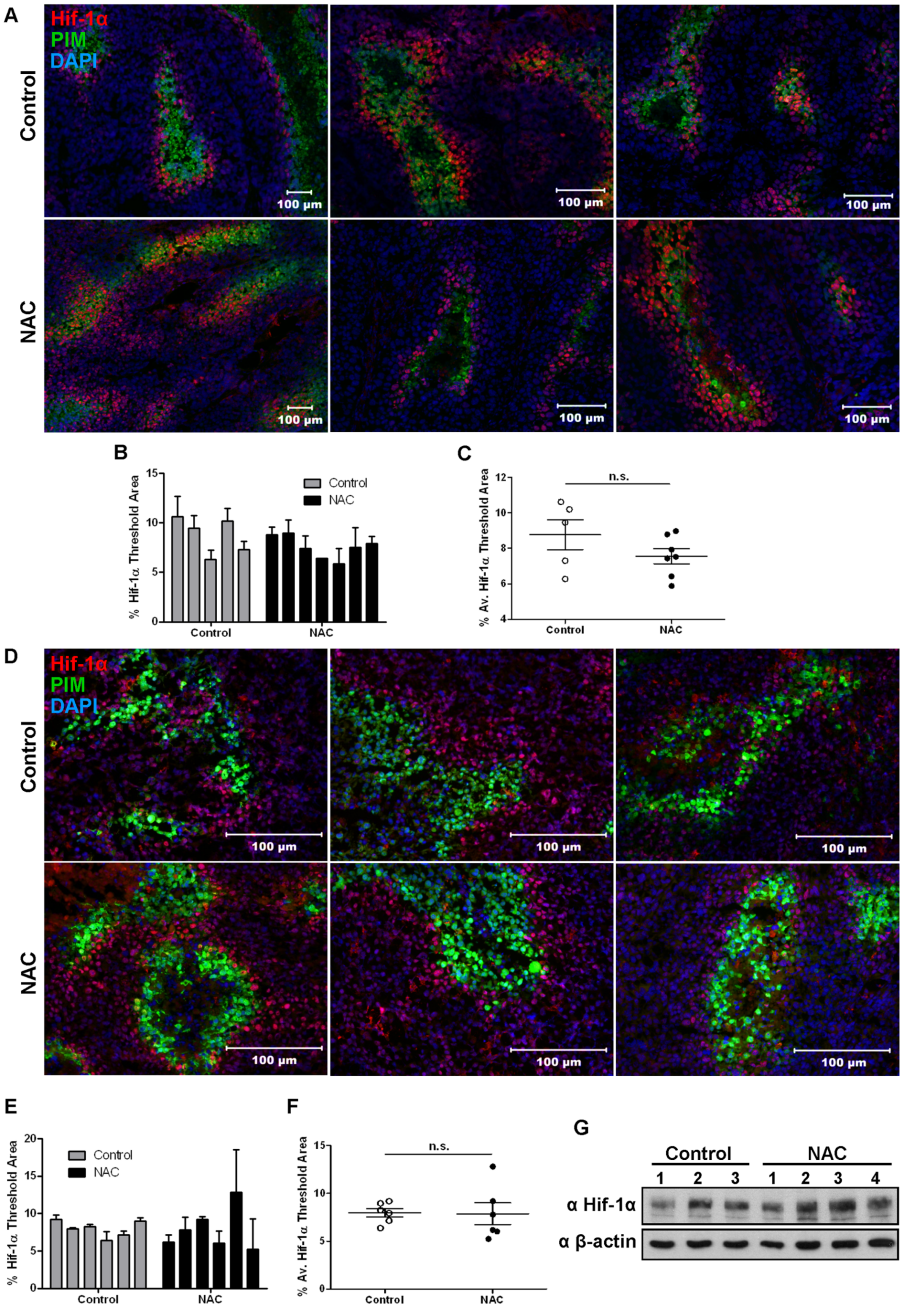
NAC treatment does not alter the hypoxic phenotype of primary tumors. Tumors from NAC-treated or control mice were stained with Hif-1α (red), PIM (green) and DAPI (blue). Five sections per tumor were stained and imaged to obtain an average percentage threshold area of Hif-1α staining determined from 5 random images per section. **A)** Representative images of PyMT endstage tumors (from Figure 3G) with Hif-1α staining values calculated for individual tumors (**B)** and averaged **(C)** (control n = 5 tumors; NAC n = 7 tumors). **D)** Representative images of EO771 endstage tumors (from Figure 3C–D) with Hif-1α staining values calculated for individual tumors (**E)** and averaged **(F)** (n = 6 tumors for all groups). Mean ± SEM; n.s. indicates not significant. **G)** EO771 tumor lysates (from Figure 3C–D) were probed for Hif-1α and β-actin (loading control) by Western blot (control n = 3 tumors; NAC n = 4 tumors).

To assess the prevalence of Hif-1α-positive cells and the overall hypoxic state of tumors from mice treated with NAC, the hypoxia probe pimonidazole (PIM) was injected before sacrificing the mice. Tumors were stained and evaluated for Hif-1α and PIM. No difference was observed in the abundance of Hif-1α positive cells in PyMT tumors from mice treated with NAC after establishment of the primary tumor compared to controls (Figure 5A–C, control n = 7; NAC n = 8; 5 sections stained and analyzed per tumor). EO771 tumors from mice pre-treated with NAC from day -7 were also similar to controls with regard to Hif-1α-positive cells (Figure 5D–F; control n = 6; NAC n = 5; 5 sections stained and analyzed per tumor). Furthermore, NAC treatment did not change the protein abundance of Hif-1α in EO771 tumor lysates compared to controls as detected by Western blot (Figure 5G). In both models, Hif-1α staining was still closely associated with hypoxic (PIM-positive) regions regardless of NAC treatment, indicating NAC treatment had no effect on the hypoxic response in primary breast tumors *in vivo*.

### NAC Treatment Promotes Metastatic Outgrowth in the EO771 Experimental Metastasis Model

**Figure 6:**
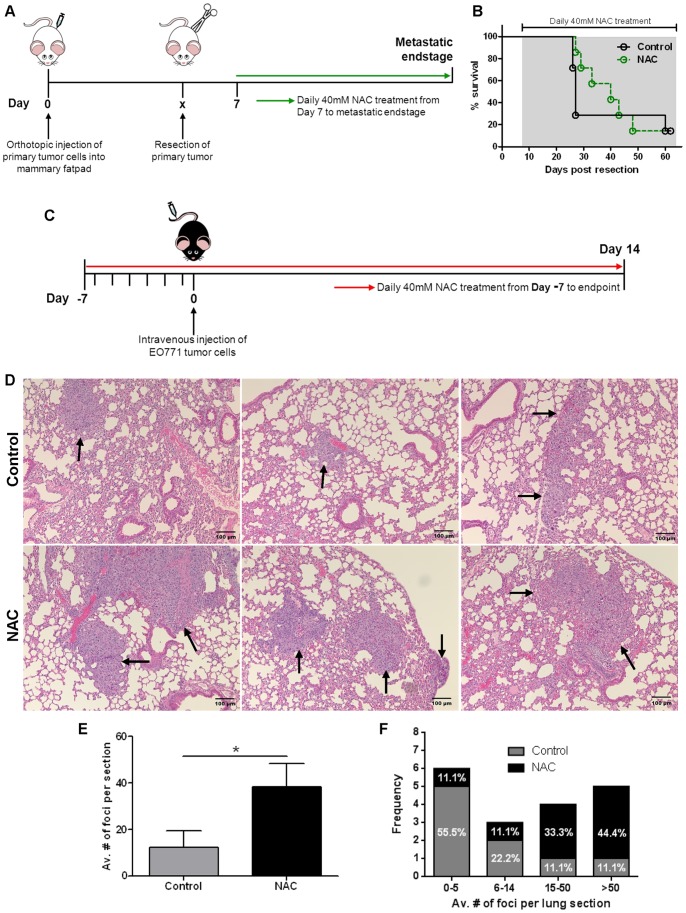
NAC treatment increases metastatic burden in an EO771 experimental metastasis model. **A)** Schematic of NAC treatment regimen for 4T1.2 primary-resection model. BALB/C mice were orthotopically injected with 4T1.2 breast tumor cells on day 0, and the primary tumor surgically resected at a volume of 35 mm^3^ (∼day 10). NAC was administered at 40 mM in the drinking water 7 days post-resection (green line) until metastatic endstage. **B)** Kaplan-Meier survival curve of mice treated as in A (n = 6 all groups; p = 0.57). p values calculated using Log-rank (Mantel-Cox) Test. **C)** Schematic of NAC treatment regimen for experimental metastasis model. NAC (40 mM) was administered in the drinking water from day -7 (red line) prior to EO771 tumor cell injection. Mice were intravenously injected with EO771 tumor cells through the tail vein on day 0, and lungs analyzed for metastatic burden at day 14. **D)** Representative histology images (H&E stained) of lungs from C, with metastatic foci indicated by black arrows. Scale bar represents 100 µm. **E)** Average number of metastatic foci per lung (average determined from 4 whole lung sections per mouse) from D (n = 9 per group). Mean ± SEM; *p<0.05; Student’s t-Test. **F)** Absolute frequency and percentage of mice with an average of between 0–5, 6–14, 15–50 and >50 metastatic foci per lung section from E.

To determine if NAC treatment had any effect on metastasis in our breast cancer mouse models, we used two different experimental methods. In the primary-resection model, established 4T1.2 breast tumors were surgically removed at a volume of 35 mm^3^, and NAC treatment started 7 days post-resection. The mice were culled when they displayed signs of metastatic disease (detailed in Materials and Methods) (Figure 6A). No difference in overall survival of NAC-treated compared to control mice was observed (Figure 6B; n = 9 per group).

In the experimental metastasis model, EO771 breast tumor cells were injected directly into the circulation of mice via the tail vein 7 days after NAC treatment was commenced. The mice were culled 14 days post-injection and metastatic foci counted in H&E sections of whole lungs (Figure 6C). Interestingly, in the experimental metastasis model, NAC treatment increased metastatic burden in the lungs compared to controls (Figure 6D–E; n = 9 in each cohort). The number as well as the size of the metastatic foci in the lungs of NAC-treated mice was also increased compared to controls. In the control group, 55.5% (5/9) had less than 5 metastatic foci per lung section, and 44.4% (4/9) had greater than 6 metastatic foci. In the NAC-treated group, only 11.1% (1/9) had less than 5 metastatic foci, while 88.9% (8/9) had greater than 6 metastatic foci per section. Furthermore, almost half of the NAC-treated mice had greater than 50 metastatic foci detected per lung section (44.4% (4/9); Figure 6F; n = 9 in each group; 4 sections scored per lung).

**Figure 7:**
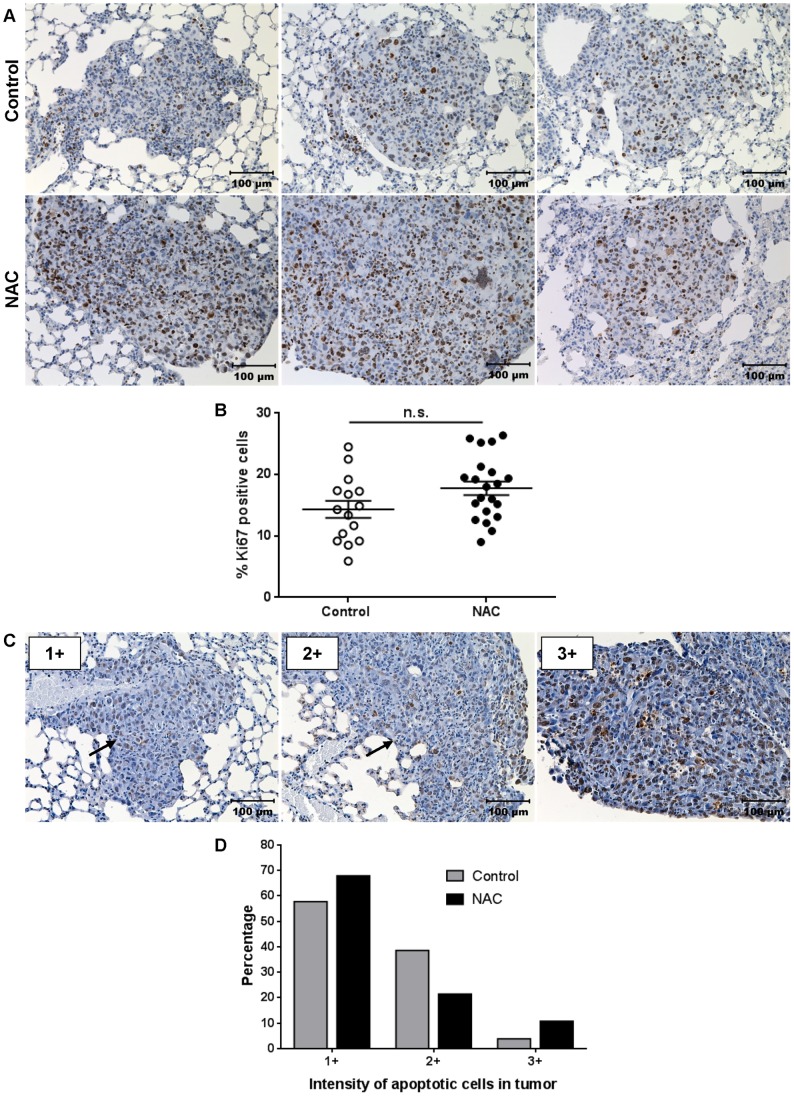
NAC treatment does not significantly effect proliferation and apoptosis in EO771 metastatic tumors. **A)** Representative images of lungs (from Figure 6D–E) stained with Ki67 (positive cells stained brown) from control and NAC-treated mice. Scale bars represent 100 µm. **B)** Percentage of Ki67 positive cells per metastatic tumor from A analyzed using the ImmunoRatio image analysis program (control n = 15 and NAC n = 21 tumors analyzed from 6 mice per group). Mean ± SEM; n.s. indicates not significant. **C)** Examples of 1+, 2+ or 3+ metastatic tumors regarding intensity of staining for apoptotic cells (positive cells stained brown) with tumors indicated by black arrows where necessary. Scale bars represent 100 µm. **D)** Percentage of metastatic tumors in control and NAC-treated mice (from Figure 6D–E) categorized as 1+, 2+ or 3+ for staining of apoptotic cells (control n = 16 and NAC n = 28 tumors analyzed from 7 mice per group).

Next, we assessed the proliferation and apoptosis in these EO771 metastatic lesions. Each metastatic tumor was analyzed separately as an independent sample, due to the limited number of metastatic tumors per lung in the control group. Metastatic tumors from NAC-treated mice had an increased percentage of Ki67 positive tumor cells compared to controls, though this was not significant (Figure 7A–B; p = 0.058; control n = 15 and NAC n = 21 tumors analyzed). Apoptosis in metastatic tumors was characterized by abundance and intensity of staining in a blinded manner into categories of 0 (no apoptosis), 1+ (little apoptosis), 2+ (some apoptosis) or 3+ (extensive apoptosis) (examples in Figure 7C). In metastatic tumors from NAC-treated mice, 67.9% were categorized as 1+, 21.4% as 2+ and 10.7% as 3+ tumors (Figure 7D). In comparison, 57.7% control tumors were categorized as 1+, 38.5% as 2+ and 3.8% as 3+ tumors. Overall, the data indicate a slight trend towards increased proliferation in tumors from NAC-treated mice.

## Discussion

### Is NAC a Potent Anti-cancer Agent in Cell and Animal Models?

NAC has long been considered a promising anti-tumorigenic agent. Only recently it has been proposed that NAC exerts its anti-tumorigenic effects through modulation of the hypoxic response signaling pathway in tumor cells. Our *in vitro* data is in accordance with a recent study demonstrating that treatment of human P493 B cells and PC3 prostate carcinoma cells with 10 mM NAC prevents Hif-1α stabilization and reduces VEGF secretion under hypoxic conditions (1% O_2_) [33]. By removing ROS, NAC treatment prevented inactivation of PHD2 under hypoxic conditions, resulting in Hif-1α degradation [33].

GSH plays a central role in the antioxidant defense against ROS, but synthesis of GSH can be limited by the availability of cysteine [41]. NAC is a precursor of GSH, and supplementation with NAC increases the levels of GSH in the body [35]. We found that GSH levels in the blood were significantly increased within 48 hours of adding NAC to the drinking water of mice. After 2 and 6 weeks of treatment, GSH levels were still increased, but this increase was no longer significant compared to controls. Yet the increased GSH levels in the blood of NAC-treated mice did not translate into significant differences in GSH levels in the tumors themselves, which could explain why we did not observe any significant anti-cancer effects on primary tumor growth.

Overall survival and primary tumor growth rates did not change after NAC treatment in multiple orthotopic, syngeneic murine models of breast cancer, regardless of the timing of NAC administration during tumorigenesis. These results are in contrast to previous data, where 40 mM NAC treatment in the drinking water inhibited primary tumor growth in both a MYC-dependent human B cell lymphoma model (P493) and TRE-LAP MYC transgenic mice overexpressing MYC in liver cells [33]. In prostate cancer on the other hand, treatment with NAC was reported to decrease ROS levels in Nkx3.1 mutant mouse prostates, but actually increase epithelial cell proliferation and expression of a pro-proliferative gene signature [53]. Treatment with NAC also reportedly hinders the effects of certain chemotherapeutic drugs, such as paclitaxel, by decreasing ROS levels to the extent that apoptosis of tumor cells is prevented [8], and alters the response of the redox-sensitive NF-κB signal transduction pathway in tumor cells targeted by doxorubicin-induced ROS [54].

These different outcomes may be explained by the various tumor models used. In our model, the ability of NAC to penetrate solid breast tumors in sufficient levels might be hindered by the poor tumor vasculature, which is not an obstacle in blood cancers such as lymphomas [4]. In Kaposi’s sarcoma, a highly vascularized human tumor, NAC treatment proved effective at inhibiting tumor growth even after the tumor mass was detectable and established [37]. NAC is also resorbed rapidly after oral administration, but due to extensive first-pass metabolism its plasma concentration and tissue availability may be reduced [41], suggesting that these NAC doses may be insufficient in poorly vascularized tumors.

Reports regarding the outcome of NAC treatment in breast cancer are limited, making interpretation of its effects in this cancer type difficult. In a xenograft MBA-MB-435 model of breast cancer, subcutaneous injections of NAC at 10 mg/kg body weight daily decreased tumor growth by promoting the production of anti-angiogenic factors such as angiostatin, resulting in vascular collapse and extensive necrosis within the tumor [36]. Oral administration of 40 mM NAC in the drinking water, calculated to equal 1 g/kg daily [33], failed to alter CD31-positive blood vessel density or necrosis in our syngeneic breast cancer models. The different route of administration of NAC in our study may be another explanation for the differences in outcome.

In the aforementioned studies, NAC has also shown effectiveness as an anti-metastatic agent, but many of these observations occur as a consequence of decreased primary tumor growth. Smaller or slower growing primary tumors are less likely to metastasize to the same extent as larger or faster growing tumors, and NAC may also inhibit metastatic tumor growth in the same manner as the primary tumor. We found that NAC treatment had no influence in the primary tumor resection model, which closely recapitulates treatment in breast cancer patients. In contrast, administration of NAC prior to intravenous tumor cell injection in an experimental metastasis model actually increased metastatic tumor burden. NAC treatment in experimental metastasis models has previously only been studied in the B16-F10 melanoma model [43]. When pre-treated with NAC and resuspended in medium supplemented with NAC, intravenous injection of B16-F10 cells into nude mice resulted in an overall decrease in lung metastases [43]. However this work did not investigate metastasis formation without NAC pre-treatment. In another report, treatment with NAC and doxorubicin acted synergistically to reduce metastastatic growth more than either drug alone [39]. Intriguingly and similar to our data, the control group treated with NAC alone (1 mg/kg intraperitoneal injections daily) had an increased number of metastases, although this was not significant [39]. The underlying mechanism of enhanced tumor growth may, in part, be explained by the subtle changes we observed in proliferation and apoptosis in metastatic tumors from control and NAC-treated mice. NAC may protect metastasized tumor cells from apoptosis by removing DNA-damaging ROS, allowing continuous proliferation upon their arrival in secondary organs and creating favourable growth conditions to increase the efficiency of the normally inefficient metastatic cascade.

Other reported effects of NAC may also contribute to the increase in metastatic burden, such as its ability to act as a vasodilator [41], [55] and to decrease neutrophil activation [41], [56], [57]. Vessel dilation may allow more tumor cells from the primary site to enter circulation, and increase the numbers of circulating tumor cells to improve the chance of metastatic colonization and outgrowth. Phagocytes including neutrophils and immunologically activated-macrophages, produce reactive oxygen intermediates to damage foreign cells [58]. NAC has been utilised to combat the neutrophil-mediated oxidant damage associated with acute lung injury [59], indicating that NAC is capable of attenuating the oxidative burst function of neutrophils. It is possible therefore that, in a cancer context, NAC diminishes the ability of phagocytes including neutrophils and macrophages to kill tumor cells.

### Is NAC a Useful Therapeutic against Cancer in Patients?

In comparison to the mixed results reported in animal studies, there is very little evidence supporting the use of NAC as a direct anti-cancer agent in the clinic. NAC has been used in cancer therapy to alleviate chemotherapy-induced toxicity evidenced in a number of studies [39], [43], [53], [60], [61], but has had limited success alone or in combination with chemotherapeutic drugs [7], [41]. In a large randomized trial (EUROSCAN) designed to investigate the effects of NAC and Vitamin A in the prevention of tumor recurrence and metastatic relapse in patients with lung or head and neck cancer, daily administration of either antioxidant for 2 years yielded no benefits within the first 2-year follow-up period [62]. Three studies conducted between 1980 and 1990, which focused on tumor-related endpoints in lung cancer patients, also showed no apparent benefit after NAC treatment [53], [63], [64]. This is true for other antioxidants as well, raising the question of the usefulness of antioxidants in general in the treatment of cancer. The SELECT trial, initiated in 2001 to test the efficacy of the antioxidants selenium and Vitamin E in the prevention of prostate cancer [65], showed that Vitamin E actually increased the risk of prostate cancer development over the time span of 10 years [66]. Antioxidants, like many other chemotherapeutic agents, may be useful in certain cancer types and patient cohorts, but not as a general treatment. The response of the tumor to hypoxia, ROS production, pro-angiogenic and pro-metastatic cues may determine the susceptibility of tumors to NAC intervention, and this needs to be investigated in different cancer models in order understand the potential applications of NAC in cancer therapy.

## Supporting Information

Figure S1 **A)** Tumor sections were H&E stained and whole sections scanned to assess overall tumor necrosis (tumors described in Figure 3G). **B)** Representative images of the data output from the ImmunoRatio program (described in Materials and Methods) for Ki67 stained lung sections (from Figure 7A-B) of control and NAC metastatic tumors. The region of interest tool was used to define the metastatic tumor area and avoid the inclusion of normal lung tissue, giving a percentage of DAB (Ki67) positive cells in each tumor.
(TIF)

## References

[1] Semenza GL (2012) Hypoxia-inducible factors: mediators of cancer progression and targets for cancer therapy. Trends Pharmacol Sci 33: 207–214. 22398146 10.1016/j.tips.2012.01.005 PMC3437546

[2] Lu X, Kang Y (2010) Hypoxia and hypoxia-inducible factors: master regulators of metastasis. Clin Cancer Res 16: 5928–5935. 20962028 10.1158/1078-0432.CCR-10-1360 PMC3005023

[3] Harris AL (2002) Hypoxia–a key regulatory factor in tumour growth. Nat Rev Cancer 2: 38–47. 11902584 10.1038/nrc704

[4] Jain RK (2005) Normalization of tumor vasculature: an emerging concept in antiangiogenic therapy. Science 307: 58–62. 15637262 10.1126/science.1104819

[5] Pries AR, Cornelissen AJ, Sloot AA, Hinkeldey M, Dreher MR, et al (2009) Structural adaptation and heterogeneity of normal and tumor microvascular networks. PLoS Comput Biol 5: e1000394. 19478883 10.1371/journal.pcbi.1000394 PMC2682204

[6] Wilson WR, Hay MP (2011) Targeting hypoxia in cancer therapy. Nat Rev Cancer 11: 393–410. 21606941 10.1038/nrc3064

[7] Moeller BJ, Richardson RA, Dewhirst MW (2007) Hypoxia and radiotherapy: opportunities for improved outcomes in cancer treatment. Cancer Metastasis Rev 26: 241–248. 17440683 10.1007/s10555-007-9056-0

[8] Rohwer N, Cramer T (2011) Hypoxia-mediated drug resistance: novel insights on the functional interaction of HIFs and cell death pathways. Drug Resist Updat 14: 191–201. 21466972 10.1016/j.drup.2011.03.001

[9] Semenza GL (1999) Regulation of mammalian O2 homeostasis by hypoxia-inducible factor 1. Annu Rev Cell Dev Biol 15: 551–578. 10611972 10.1146/annurev.cellbio.15.1.551

[10] Wang GL, Jiang BH, Rue EA, Semenza GL (1995) Hypoxia-inducible factor 1 is a basic-helix-loop-helix-PAS heterodimer regulated by cellular O2 tension. Proc Natl Acad Sci U S A 92: 5510–5514. 7539918 10.1073/pnas.92.12.5510 PMC41725

[11] Jiang BH, Semenza GL, Bauer C, Marti HH (1996) Hypoxia-inducible factor 1 levels vary exponentially over a physiologically relevant range of O2 tension. Am J Physiol 271: C1172–1180. 8897823 10.1152/ajpcell.1996.271.4.C1172

[12] Huang LE, Gu J, Schau M, Bunn HF (1998) Regulation of hypoxia-inducible factor 1alpha is mediated by an O2-dependent degradation domain via the ubiquitin-proteasome pathway. Proc Natl Acad Sci U S A 95: 7987–7992. 9653127 10.1073/pnas.95.14.7987 PMC20916

[13] Kallio PJ, Wilson WJ, O’Brien S, Makino Y, Poellinger L (1999) Regulation of the hypoxia-inducible transcription factor 1alpha by the ubiquitin-proteasome pathway. J Biol Chem 274: 6519–6525. 10037745 10.1074/jbc.274.10.6519

[14] Salceda S, Caro J (1997) Hypoxia-inducible factor 1alpha (HIF-1alpha) protein is rapidly degraded by the ubiquitin-proteasome system under normoxic conditions. Its stabilization by hypoxia depends on redox-induced changes. J Biol Chem 272: 22642–22647. 9278421 10.1074/jbc.272.36.22642

[15] Pugh CW, Ratcliffe PJ (2003) Regulation of angiogenesis by hypoxia: role of the HIF system. Nat Med 9: 677–684. 12778166 10.1038/nm0603-677

[16] Tian H, McKnight SL, Russell DW (1997) Endothelial PAS domain protein 1 (EPAS1), a transcription factor selectively expressed in endothelial cells. Genes Dev 11: 72–82. 9000051 10.1101/gad.11.1.72

[17] Wiesener MS, Turley H, Allen WE, Willam C, Eckardt KU, et al (1998) Induction of endothelial PAS domain protein-1 by hypoxia: characterization and comparison with hypoxia-inducible factor-1alpha. Blood 92: 2260–2268. 9746763

[18] Semenza GL (2010) Defining the role of hypoxia-inducible factor 1 in cancer biology and therapeutics. Oncogene 29: 625–634. 19946328 10.1038/onc.2009.441 PMC2969168

[19] Majmundar AJ, Wong WJ, Simon MC (2010) Hypoxia-inducible factors and the response to hypoxic stress. Mol Cell 40: 294–309. 20965423 10.1016/j.molcel.2010.09.022 PMC3143508

[20] Bruick RK, McKnight SL (2001) A conserved family of prolyl-4-hydroxylases that modify HIF. Science 294: 1337–1340. 11598268 10.1126/science.1066373

[21] Epstein AC, Gleadle JM, McNeill LA, Hewitson KS, O’Rourke J, et al (2001) C. elegans EGL-9 and mammalian homologs define a family of dioxygenases that regulate HIF by prolyl hydroxylation. Cell 107: 43–54. 11595184 10.1016/s0092-8674(01)00507-4

[22] Yu AY, Shimoda LA, Iyer NV, Huso DL, Sun X, et al (1999) Impaired physiological responses to chronic hypoxia in mice partially deficient for hypoxia-inducible factor 1alpha. J Clin Invest 103: 691–696. 10074486 10.1172/JCI5912 PMC408131

[23] Ivan M, Kondo K, Yang H, Kim W, Valiando J, et al (2001) HIFalpha targeted for VHL-mediated destruction by proline hydroxylation: implications for O2 sensing. Science 292: 464–468. 11292862 10.1126/science.1059817

[24] Jaakkola P, Mole DR, Tian YM, Wilson MI, Gielbert J, et al (2001) Targeting of HIF-alpha to the von Hippel-Lindau ubiquitylation complex by O2-regulated prolyl hydroxylation. Science 292: 468–472. 11292861 10.1126/science.1059796

[25] Maxwell PH, Wiesener MS, Chang GW, Clifford SC, Vaux EC, et al (1999) The tumour suppressor protein VHL targets hypoxia-inducible factors for oxygen-dependent proteolysis. Nature 399: 271–275. 10353251 10.1038/20459

[26] Lee K, Qian DZ, Rey S, Wei H, Liu JO, et al (2009) Anthracycline chemotherapy inhibits HIF-1 transcriptional activity and tumor-induced mobilization of circulating angiogenic cells. Proc Natl Acad Sci U S A 106: 2353–2358. 19168635 10.1073/pnas.0812801106 PMC2650160

[27] Qian DZ, Kachhap SK, Collis SJ, Verheul HM, Carducci MA, et al (2006) Class II histone deacetylases are associated with VHL-independent regulation of hypoxia-inducible factor 1 alpha. Cancer Res 66: 8814–8821. 16951198 10.1158/0008-5472.CAN-05-4598

[28] Isaacs JS, Jung YJ, Mimnaugh EG, Martinez A, Cuttitta F, et al (2002) Hsp90 regulates a von Hippel Lindau-independent hypoxia-inducible factor-1 alpha-degradative pathway. J Biol Chem 277: 29936–29944. 12052835 10.1074/jbc.M204733200

[29] Thomas GV, Tran C, Mellinghoff IK, Welsbie DS, Chan E, et al (2006) Hypoxia-inducible factor determines sensitivity to inhibitors of mTOR in kidney cancer. Nat Med 12: 122–127. 16341243 10.1038/nm1337

[30] Liu M, Howes A, Lesperance J, Stallcup WB, Hauser CA, et al (2005) Antitumor activity of rapamycin in a transgenic mouse model of ErbB2-dependent human breast cancer. Cancer Res 65: 5325–5336. 15958580 10.1158/0008-5472.CAN-04-4589

[31] Luwor RB, Lu Y, Li X, Mendelsohn J, Fan Z (2005) The antiepidermal growth factor receptor monoclonal antibody cetuximab/C225 reduces hypoxia-inducible factor-1 alpha, leading to transcriptional inhibition of vascular endothelial growth factor expression. Oncogene 24: 4433–4441. 15806152 10.1038/sj.onc.1208625

[32] Kummar S, Raffeld M, Juwara L, Horneffer Y, Strassberger A, et al (2011) Multihistology, target-driven pilot trial of oral topotecan as an inhibitor of hypoxia-inducible factor-1alpha in advanced solid tumors. Clin Cancer Res 17: 5123–5131. 21673063 10.1158/1078-0432.CCR-11-0682 PMC3149769

[33] Gao P, Zhang H, Dinavahi R, Li F, Xiang Y, et al (2007) HIF-dependent antitumorigenic effect of antioxidants in vivo. Cancer Cell 12: 230–238. 17785204 10.1016/j.ccr.2007.08.004 PMC2084208

[34] Brok J, Buckley N, Gluud C (2006) Interventions for paracetamol (acetaminophen) overdose. Cochrane Database Syst Rev: CD003328. 10.1002/14651858.CD003328.pub2 16625578

[35] Millea PJ (2009) N-acetylcysteine: multiple clinical applications. Am Fam Physician 80: 265–269. 19621836

[36] Agarwal A, Munoz-Najar U, Klueh U, Shih SC, Claffey KP (2004) N-acetyl-cysteine promotes angiostatin production and vascular collapse in an orthotopic model of breast cancer. Am J Pathol 164: 1683–1696. 15111315 10.1016/S0002-9440(10)63727-3 PMC1615662

[37] Albini A, Morini M, D’Agostini F, Ferrari N, Campelli F, et al (2001) Inhibition of angiogenesis-driven Kaposi’s sarcoma tumor growth in nude mice by oral N-acetylcysteine. Cancer Res 61: 8171–8178. 11719447

[38] Kelloff GJ, Crowell JA, Boone CW, Steele VE, Lubet RA, et al.. (1994) Clinical development plan: N-Acetyl-l-cysteine. J Cell Biochem Suppl 20: 63–73. 7616754

[39] De Flora S, D’Agostini F, Masiello L, Giunciuglio D, Albini A (1996) Synergism between N-acetylcysteine and doxorubicin in the prevention of tumorigenicity and metastasis in murine models. Int J Cancer 67: 842–848. 8824557 10.1002/(SICI)1097-0215(19960917)67:6<842::AID-IJC14>3.0.CO;2-3

[40] D’Agostini F, Bagnasco M, Giunciuglio D, Albini A, De Flora S (1998) Inhibition by oral N-acetylcysteine of doxorubicin-induced clastogenicity and alopecia, and prevention of primary tumors and lung micrometastases in mice. Int J Oncol 13: 217–224. 9664114 10.3892/ijo.13.2.217

[41] Aitio ML (2006) N-acetylcysteine – passe-partout or much ado about nothing? Br J Clin Pharmacol 61: 5–15. 16390346 10.1111/j.1365-2125.2005.02523.x PMC1884975

[42] Calvani M, Comito G, Giannoni E, Chiarugi P (2012) Time-dependent stabilization of hypoxia inducible factor-1alpha by different intracellular sources of reactive oxygen species. PLoS One 7: e38388. 23144690 10.1371/journal.pone.0038388 PMC3483303

[43] Albini A, D’Agostini F, Giunciuglio D, Paglieri I, Balansky R, et al (1995) Inhibition of invasion, gelatinase activity, tumor take and metastasis of malignant cells by N-acetylcysteine. Int J Cancer 61: 121–129. 7705924 10.1002/ijc.2910610121

[44] Shimojo Y, Akimoto M, Hisanaga T, Tanaka T, Tajima Y, et al.. (2012) Attenuation of reactive oxygen species by antioxidants suppresses hypoxia-induced epithelial-mesenchymal transition and metastasis of pancreatic cancer cells. Clin Exp Metastasis. 10.1007/s10585-012-9519-8 22833345

[45] Wong CS, Sceneay J, House CM, Halse HM, Liu MC, et al (2012) Vascular normalization by loss of Siah2 results in increased chemotherapeutic efficacy. Cancer Res 72: 1694–1704. 22354750 10.1158/0008-5472.CAN-11-3310

[46] Sceneay J, Chow MT, Chen A, Halse HM, Wong CSF, et al.. (2012) Primary Tumor Hypoxia Recruits CD11b+/Ly6Cmed/Ly6G+ Immune Suppressor Cells and Compromises NK Cell Cytotoxicity in the Premetastatic Niche. Cancer Research. 10.1158/0008-5472.CAN-11-3873 22751463

[47] Casey AE, Laster WR Jr, Ross GL (1951) Sustained enhanced growth of carcinoma EO771 in C57 black mice. Proc Soc Exp Biol Med 77: 358–362. 14854049 10.3181/00379727-77-18779

[48] Lelekakis M, Moseley JM, Martin TJ, Hards D, Williams E, et al (1999) A novel orthotopic model of breast cancer metastasis to bone. Clin Exp Metastasis 17: 163–170. 10411109 10.1023/a:1006689719505

[49] Moller A, House CM, Wong CS, Scanlon DB, Liu MC, et al (2009) Inhibition of Siah ubiquitin ligase function. Oncogene 28: 289–296. 18850011 10.1038/onc.2008.382 PMC3000903

[50] Bidwell BN, Slaney CY, Withana NP, Forster S, Cao Y, et al.. (2012) Silencing of Irf7 pathways in breast cancer cells promotes bone metastasis through immune escape. Nat Med. 10.1038/nm.2830 22820642

[51] Young RJ, Moller A (2010) Immunohistochemical detection of tumour hypoxia. Methods Mol Biol 611: 151–159. 19960329 10.1007/978-1-60327-345-9_12

[52] Tuominen VJ, Ruotoistenmaki S, Viitanen A, Jumppanen M, Isola J (2010) ImmunoRatio: a publicly available web application for quantitative image analysis of estrogen receptor (ER), progesterone receptor (PR), and Ki-67. Breast Cancer Res 12: R56. 20663194 10.1186/bcr2615 PMC2949645

[53] Morgan LR, Donley PJ, Harrison EF, Hunter HL (1982) Protective effect of N-acetylcysteine on the urotoxicity produced by oxazaphosphorine without interference with anticancer activity. Eur J Cancer Clin Oncol 18: 113–114. 7200891 10.1016/0277-5379(82)90035-9

[54] Pastorino U, Chiesa G, Infante M, Soresi E, Clerici M, et al (1991) Safety of high-dose vitamin A. Randomized trial on lung cancer chemoprevention. Oncology 48: 131–137. 1671795 10.1159/000226912

[55] Zafarullah M, Li WQ, Sylvester J, Ahmad M (2003) Molecular mechanisms of N-acetylcysteine actions. Cell Mol Life Sci 60: 6–20. 12613655 10.1007/s000180300001 PMC11138873

[56] Allegra L, Dal Sasso M, Bovio C, Massoni C, Fonti E, et al (2002) Human neutrophil oxidative bursts and their in vitro modulation by different N-acetylcysteine concentrations. Arzneimittelforschung 52: 669–676. 12404881 10.1055/s-0031-1299949

[57] Heller AR, Groth G, Heller SC, Breitkreutz R, Nebe T, et al (2001) N-acetylcysteine reduces respiratory burst but augments neutrophil phagocytosis in intensive care unit patients. Crit Care Med 29: 272–276. 11246305 10.1097/00003246-200102000-00009

[58] Nathan C, Shiloh MU (2000) Reactive oxygen and nitrogen intermediates in the relationship between mammalian hosts and microbial pathogens. Proc Natl Acad Sci U S A 97: 8841–8848. 10922044 10.1073/pnas.97.16.8841 PMC34021

[59] Davreux CJ, Soric I, Nathens AB, Watson RW, McGilvray ID, et al (1997) N-acetyl cysteine attenuates acute lung injury in the rat. Shock 8: 432–438. 9421857

[60] Mantovani G, Maccio A, Madeddu C, Mura L, Gramignano G, et al (2003) Antioxidant agents are effective in inducing lymphocyte progression through cell cycle in advanced cancer patients: assessment of the most important laboratory indexes of cachexia and oxidative stress. J Mol Med 81: 664–673. 12928788 10.1007/s00109-003-0476-1

[61] Sjoo F, Aschan J, Barkholt L, Hassan Z, Ringden O, et al (2003) N-acetyl-L-cysteine does not affect the pharmacokinetics or myelosuppressive effect of busulfan during conditioning prior to allogeneic stem cell transplantation. Bone Marrow Transplant 32: 349–354. 12900770 10.1038/sj.bmt.1704143

[62] van Zandwijk N, Dalesio O, Pastorino U, de Vries N, van Tinteren H (2000) EUROSCAN, a randomized trial of vitamin A and N-acetylcysteine in patients with head and neck cancer or lung cancer. For the EUropean Organization for Research and Treatment of Cancer Head and Neck and Lung Cancer Cooperative Groups. J Natl Cancer Inst 92: 977–986. 10861309 10.1093/jnci/92.12.977

[63] Loehrer PJ Sr, Birch R, Kramer BS, Greco FA, Einhorn LH (1986) Ifosfamide plus N-acetylcysteine in the treatment of small cell and non-small cell carcinoma of the lung: a Southeastern Cancer Study Group Trial. Cancer Treat Rep 70: 919–920. 3013402

[64] Maasilta P, Holsti LR, Blomqvist P, Kivisaari L, Mattson K (1992) N-acetylcysteine in combination with radiotherapy in the treatment of non-small cell lung cancer: a feasibility study. Radiother Oncol 25: 192–195. 1335155 10.1016/0167-8140(92)90267-x

[65] Klein EA, Thompson IM, Lippman SM, Goodman PJ, Albanes D, et al (2001) SELECT: the next prostate cancer prevention trial. Selenum and Vitamin E Cancer Prevention Trial. J Urol 166: 1311–1315. 11547064 10.1016/s0022-5347(05)65759-x

[66] Klein EA, Thompson IM Jr, Tangen CM, Crowley JJ, Lucia MS, et al (2011) Vitamin E and the risk of prostate cancer: the Selenium and Vitamin E Cancer Prevention Trial (SELECT). JAMA 306: 1549–1556. 21990298 10.1001/jama.2011.1437 PMC4169010

