# Supplementary figures and images for: The Antioxidant N-Acetylcysteine Prevents HIF-1 Stabilization under Hypoxia In Vitro but Does Not Affect Tumorigenesis in Multiple Breast Cancer Models In Vivo
Source: PLoS One. 2013 Jun 20;8(6):e66388. doi: 10.1371/journal.pone.0066388 (PMC3688768; doi:10.1371/journal.pone.0066388)

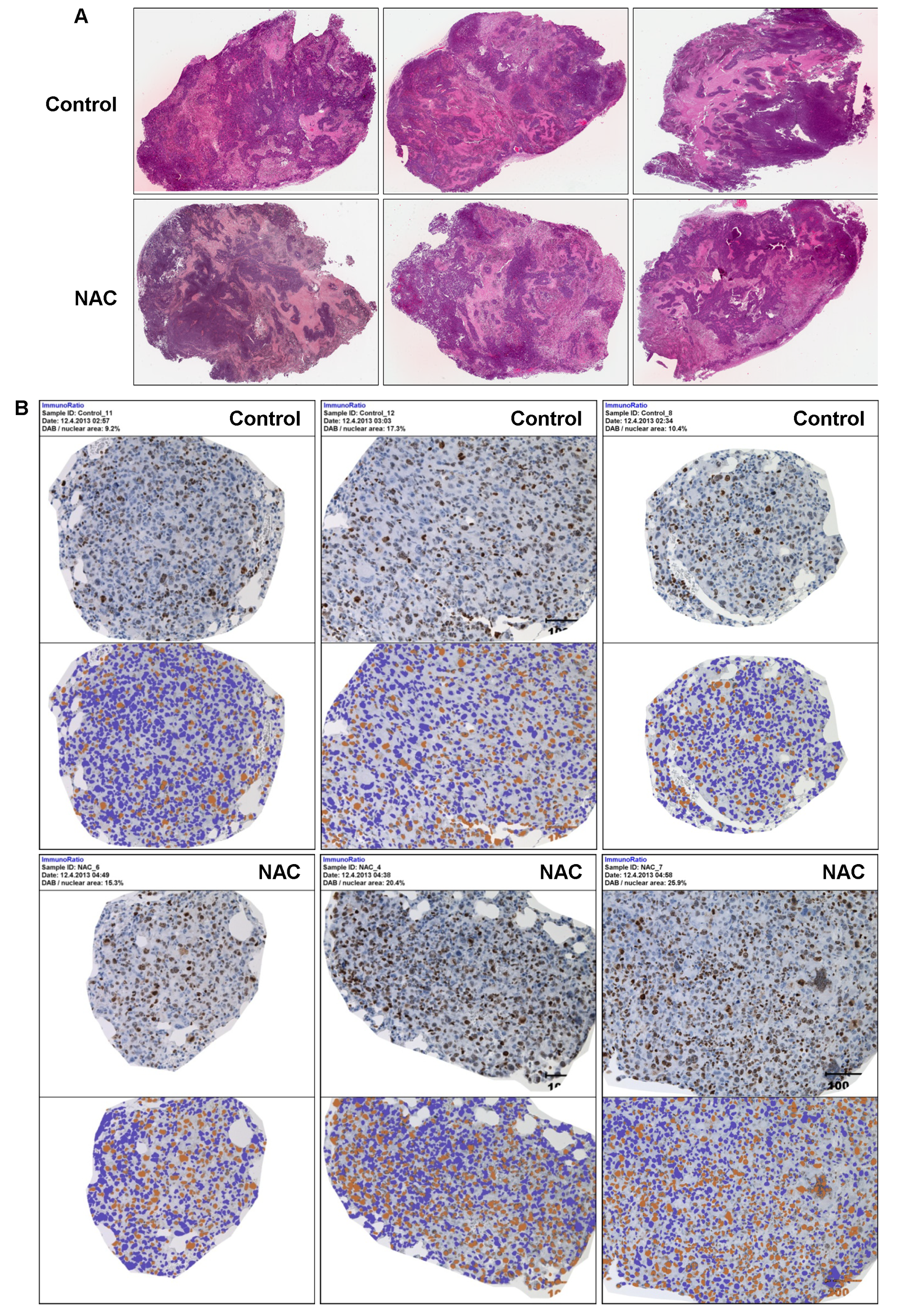

Supplement: Figure S1 — A) Tumor sections were H&E stained and whole sections scanned to assess overall tumor necrosis (tumors described in Figure 3G). B) Representative images of the data output from the ImmunoRatio program (described in Materials and Methods) for Ki67 stained lung sections (from Figure 7A-B) of control and NAC metastatic tumors. The region of interest tool was used to define the metastatic tumor area and avoid the inclusion of normal lung tissue, giving a percentage of DAB (Ki67) positive cells in each tumor. (TIF) [file pone.0066388.s001.tif]
